# Enhancing the authenticity of animal by-products: harmonization of DNA extraction methods from novel ingredients

**DOI:** 10.3389/fchem.2024.1350433

**Published:** 2024-02-20

**Authors:** Andreia Filipa-Silva, Raquel Castro, Mariana Rebelo, Maria J. Mota, André Almeida, Luísa M. P. Valente, Sónia Gomes

**Affiliations:** ^1^ CIIMAR/CIMAR-LA, Centro Interdisciplinar de Investigação Marinha e Ambiental, Universidade do Porto, Matosinhos, Portugal; ^2^ ICBAS, Instituto de Ciências Biomédicas Abel Salazar, Universidade do Porto, Porto, Portugal; ^3^ SORGAL, Sociedade de Óleos e Rações, S.A., São João de Ovar, Portugal; ^4^ SAVINOR - Sociedade Avícola do Norte S.A., Trofa, Portugal; ^5^ SEBOL, Comércio e Indústria do Sebo, S.A., Loures, Portugal; ^6^ ITS, Indústria Transformadora de Subprodutos, S.A., Coruche, Portugal

**Keywords:** animal feed ingredients, processed by-products, DNA extraction, PCR, swine, authenticity

## Abstract

**Introduction:** The increasing global pressure to explore alternative protein sources derived from animal by-products has opened-up opportunities, but it has also created the need to assess their compliance with labelling statements, to ensure consumer’s trust in the composition of both feed and food products. Assessing the authenticity of highly processed animal by-products, particularly within the rapidly expanding Halal food market, presents a significant challenge due to the lack of robust and standardized methodologies. However, the success of DNA based authenticity system is highly dependent on the extracted DNA quantity, quality, and purity ratios from heterogeneous matrices.

**Material and methods:** In this work, nine DNA extraction methods were tested on selected processed animal by-products with high-value and interest for the feed industry: meals from poultry meat, blood and feather, and hydrolysates from swine meat and bone, fish, and black soldier fly. The proposed DNA extraction methods are developed to specifically target swine-specific mitochondrial region, as a case study.

**Results and discussion:** Both the conventional CTAB method and the commercial kits, specifically Invisorb^®^ Spin Tissue Mini and NucleoSpin™ Food, demonstrated superior extraction efficiency and quality ratios. Nevertheless, commercial kits enabled faster detection in comparison to the conventional methods. The absence of swine DNA was successfully validated and confirmed in all animal meals and hydrolysates that did not contain swine in their composition beforehand, demonstrating their compliance with the Halal market requirements.

## Highlights

• Harmonization of DNA extraction methods from highly processed animal by-products.
• CTAB-based method, Invisorb^®^ Spin Tissue Mini and NucleoSpin™ Food demonstrated high extraction efficiency and quality ratios.
• Species-specific PCR-based method have been studied to validate the presence of swine amplicon in highly processed ingredients.
• Sensitive, rapid, and working protocol applied for novel feed ingredients authentication issues.

## 1 Introduction

The Food and Agriculture Organization (FAO) of the United Nations estimates that over 1.3 billion tons of food are wasted every year (FAO, 2014), resulting in the generation of enormous volumes of wastes and by-products, thus contributing to increased environmental pollution. As an under-exploited raw material rich in bioactive compounds such as polyphenols, antioxidants, and minerals, novel strategies and initiatives have been proposed and implemented for the effective management and valorization of these wastes and by-products. Among the strategies is the development of sustainable novel ingredients for animal feed, such as insects (Basto et al., 2023), algae (Ferreira et al., 2022), fungi (Harikrishnan et al., 2011), bacteria (Biswas et al., 2020), and by-products (Faustino et al., 2019), not only as a novel protein source but also playing an essential role in minimizing waste and enhancing the overall circular economy of the agrifood value chain. However, as the demand for these ingredients rises, and consumer’s trust on feed and food composition increases, this approach becomes crucial to assess compliance with labelling statements (Cantillo et al., 2021; Nguyen et al., 2022). The authenticity strategy involves ensuring that the ingredient composition, production processes and practices, technology, and genetic identity are accurately reflected on the label (Charlebois et al., 2016). Despite ongoing efforts to address this matter, food fraud involving cases of dilution (Taylor, 2019), substitution (Premanandh, 2013), and mislabelling (Filonzi et al., 2010; Cawthorn et al., 2013), remains a significant challenge for food producers, retailers, and regulatory authorities (Premanandh and Bin Salem, 2017).

Mislabelling not only leads to commercial fraud but also has potential health implications, especially for individuals with undisclosed allergens. Moreover, the information provided to consumers plays a significant role in shaping their food choices, often influenced by lifestyles factors like vegetarianism or religious practices such as Judaism and Islamism, where the absence of swine meat is essential (Rohman and Che Man, 2012; Malkawi, 2014; Nguyen et al., 2022). For instance, one of the key issues concerning authenticity is the adulteration of meat species in food products. In Halal food market, projected to grow by up to 20% in the coming years, there arises a pressing demand for monitoring and control of the authenticity of halal products. This need is especially critical for highly-processed food items and ingredients used in animal feed, to promote consumer confidence within the sector while also upholding food safety and security standards.

The DNA-based methodologies are a reliable means of tracking food composition, since DNA is a stable molecule that is independent of the variable conditions, unlike proteins. Consequently, DNA-based approaches serve as an ideal for ensuring the authenticity of complex and highly processed food matrices (Mafra et al., 2008; Gomes et al., 2018; Zia et al., 2020). Most DNA-based techniques employed for species identification involve the targeted amplification of one or more DNA fragments using the Polymerase Chain Reaction (PCR). This method is characterized by its rapidity, simplicity, sensitivity, and specificity (Mafra et al., 2008; Doosti et al., 2014; Sajali et al., 2018; Yao et al., 2022).

A crucial factor in feed ingredients analysis and PCR amplification is the quality of the extracted DNA (Särkinen et al., 2012). Two approaches for DNA extraction are commonly employed: conventional methods and commercialized extraction kits. Conventional methods are often cost-effective, yield higher amounts of DNA, and can be easily optimized to suit the specific requirements of the matrices. However, they may involve the use of toxic chemicals and be time-consuming. On the other hand, commercial kits are generally easier and faster to use but tend to be more expensive and yield less DNA (Sajali et al., 2018). Significant advancements have been achieved in refining DNA extraction methods for PCR-based analysis of diverse and complex matrices such as maize meals (Di Pinto et al., 2007), soybean derived food products (Mafra et al., 2008), honey (Soares et al., 2015), gelatine (Demirhan et al., 2012), olive oil (Gomes et al., 2018), fish muscle tissue (Cawthorn et al., 2011), canned tuna (Chapela et al., 2007) and other processed seafood and meat products (Soares et al., 2013; Karabasanavar et al., 2014; Yin et al., 2020). Although previous studies have shown the relevance of investigating the authenticity of processed products using molecular tools for DNA-based certification, there is a dearth of standardized and effective extraction methods for novel feed ingredients. Therefore, this study aimed to investigate the efficacity of nine different extraction procedures on both the quantity and quality of DNA isolated from selected novel processed animal by-products with high interest for the feed industry (animal meals and hydrolysates). Moreover, this study assessed the extracted DNA´s suitability for PCR amplification of swine DNA to ascertain its potential to meet the requirements of the “halal safe” market.

## 2 Materials and methods

### 2.1 Sampling

Ten processed meals from poultry meat (5 samples from different suppliers), blood (1 sample from one supplier) and feather (1 sample from 1 supplier) and 3 hydrolysates from swine meat and bone, fish and black soldier (1 sample of each from 1 supplier) were selected based on their relevance for the feed industry (Table 1). One swine sausage (positive) and 1 fennel leaf (negative) were used as controls (Table 1).

**TABLE 1:** List of novel aquafeed ingredients used in this study: Poultry Meat Meal (5 samples from different suppliers); Poultry Blood Meal (1 sample); Feather Meal (1 sample); and Hydrolysates (1 from Meat and Bone Swine, 1 from Fish, 1 from *Black Soldier* fly). Swine sausage (*Sus scrofa domesticus*) was used as positive control and fennel leaf (*Foeniculum vulgare*) as negative control.

Samples	
**Meals**	Poultry Meat Meal 1 (PMM1) ^a^
	Poultry Meat Meal 2 (PMM2) ^a^
	Poultry Meat Meal 3 (PMM3) ^a^
	Poultry Meat Meal 4 (PMM4)^a^
	Poultry Meat Meal 5 (PMM5) ^a^
	Poultry Blood Meal (PBM) ^a^
	Feather Meal (FM) ^a^
**Hydrolysates**	Meat and Bone Swine Hydrolysate (MBSH) ^b^
	Fish Hydrolysate (FH) ^b^
	*Black Soldier* fly Hydrolysate (BSH) ^b^
**Controls**	Swine sausage (*Sus scrofa domesticus*) - Positive control (PC) ^c^
	Fennel leaf (*Foeniculum vulgare*) - Negative control (NC) ^c^

^a^ —Poultry meat meal 1 (PMM1), Poultry meat meal 2 (PMM2), Poultry meat meal 3 (PMM3), Poultry meat meal 4 (PMM4), Poultry meat meal 5 (PMM5), Poultry Blood meal (PBM), Feather meal (FM): SAVINOR, SA., Portugal.

^b^ —Meat and bone swine hydrolysate (MBSH), Fish hydrolysate (FH), *Black soldier fly* hydrolysate (BSH), ETSA, group, Portugal.

^c^ —Swine sausage (*Sus scrofa domesticus*) - Positive control (PC) and Fennel leaf (*Foeniculum vulgare*) - Negative control (NC) were purchased in a local market.

Nine different DNA extraction protocols were tested in all samples, including commercial kits and conventional based-methods: a) CTAB-based method (cetyltrimethylammonium bromide) (Calbiochem, Darmstadt, Germany) (Doyle and Doyle, 1987), with some modifications reported by Azevedo-Nogueira et al. (2020); b) Modified CTAB-based method with an initial step of homogenization with liquid nitrogen (CTAB N*); c) Modified Wizard-CTAB method for complex matrices described by Aksoy and Sönmezoğlu (2022); d) Modified Wizard method with an Initial step of homogenization of the samples in liquid nitrogen (Modified Wizard-CTAB N); e) ZymoBIOMICS™ DNA Miniprep (Zymo Research Corp., CA, United States); f) Quick-DNA™ Miniprep Plus (Zymo Research Corp., CA, United States); g) Invisorb^®^ Spin Tissue Mini Invisorb^®^; h) Invisorb Spin Blood Mini (Invitek, Berlin, Germany); and i) NucleoSpin™ Food (Macherey-Nagel, Düren, Germany) (Table 2). The extractions were performed at least in duplicate assays using 60–200 mg of each sample. The extract DNAs were kept at −20°C until further analysis.

**TABLE 2:** Different DNA extraction methods employed in this study.

DNA extraction method	Kit/conventional	Supplier	References
CTAB-based method (CTAB)	Conventional	-	Doyle and Doyle, 1987; Azevedo-Nogueira et al., 2020
Modified CTAB-based method (CTAB N)^a^		-	Azevedo-Nogueira et al. (2020)
Modified Wizard-CTAB		-	Aksoy and Sönmezoğlu (2022)
Modified Wizard-CTAB N^a^		-	
ZymoBIOMICS™ DNA Miniprep	Kit	Zymo Research	-
Quick-DNA™ Miniprep Plus			-
Invisorb^®^ Spin Tissue Mini		Invitek	-
Invisorb^®^ Spin Blood Mini			-
NucleoSpin™ Food		Macherey-Nagel	-

^a^ N—Initial grinding of the samples with a mortar and pestle in liquid nitrogen.

### 2.2 CTAB-based method (CTAB)/Modified CTAB-based method (CTAB N*)

The DNA extraction from highly processed samples followed the procedure described by Doyle and Doyle (1987) with minor modifications as described by Azevedo-Nogueira et al. (2020). Briefly, to approximately 60 mg of sample, 750 µL of CTAB-extraction buffer pre-heated at 65°C (20 mM EDTA; 100 mM Tris-HCl, pH 8.0; 1.4 M NaCl; 2% w/v CTAB; 2% w/v PVP (polyvinylpyrrolidone) was added. For the Modified CTAB-based method (CTAB N*), initial grinding of the sample in liquid nitrogen was conducted prior to the lysis step. The mixture was then mixed by inversion and incubated in a water bath for 30 min at 65°C with stirring every 5 min. To the previous suspension, 750 µL of chloroform-isoamyl alcohol (24:1) at −20°C were added. The mixture was centrifuged at 17,000 g for 5 min at 4°C, and the upper phase transferred to a new tube and incubated with 10 µL of RNase A (PanReac AppliChem, Darmstadt, Germany) (100 μg/mL) at 37°C for 30 min. The mixture was mixed by inversion with 0.6 volume parts of isopropanol at −20°C, and the DNA was left for precipitation overnight at −20°C. The following day, the mixture was centrifuged at 5,000 g for 5 min at 4°C and the supernatant was discarded. The pellet was then washed with 750 µL of 70% ethanol at −20°C. After centrifugation (5 min, 5,000 g, 4°C), the supernatant was carefully discarded. The pellet was dried and resuspended in 70 µL of RNase/DNase free water (Cleaver Scientific, Rugby, United Kingdom).

### 2.3 Modified Wizard-CTAB/Modified Wizard-CTAB N method

For all samples, DNA was prepared using the protocol described by Aksoy and Sönmezoğlu (2022). For the Modified Wizard-CTAB N, initial grinding of the sample in liquid nitrogen was conducted prior to the lysis step.

### 2.4 ZymoBIOMICS™ DNA Miniprep, Quick-DNA™ Miniprep Plus, Invisorb^®^ Spin Tissue Mini, Invisorb Spin Blood Mini, and NucleoSpin™ Food

DNA extractions using the ZymoBIOMICS™ DNA Miniprep, Quick-DNA™ Miniprep Plus, Invisorb^®^ Spin Tissue Mini, Invisorb^®^ Spin Blood Mini, and NucleoSpin™ Food commercial kits were performed according to the manufacturer’s instructions with some minor modifications. Briefly, for the ZymoBIOMICS™ DNA Miniprep kit, the initial lysis step was replaced by the grinding of the samples with a mortar and pestle in liquid nitrogen. For the NucleoSpin™ Food Kit, following incubation with the lysis buffer, the homogenized was incubated with 10 µL RNase A (PanReac AppliChem, Chicago, United States) (20 mg/mL) for an additional 30 min at room temperature.

### 2.5 DNA concentration, purity, and integrity

DNA concentration and purity were estimated using DeNovix DS-11 FX (DeNovix Inc., Wilmington, United States). The concentration of extracted DNA was assessed by measuring the absorbance of the samples at A260 nm. Quality/purity was determined by analysing the A_260_/A_280_ ratio. DNA integrity was evaluated by electrophoresis in a 0.8% (w/v) agarose gel (NZYtech, Lisboa, Portugal) in 1 × TAE buffer (Tris-acetate-EDTA) (NZYtech, Lisboa, Portugal) stained with Green®Safe Premium nucleic acid stain (NZYtech, Lisboa, Portugal), and visualized under UV light using a Gel Doc XR+ (Bio-Rad Lab, Hercules, United States) and Quantity One software^®^.

### 2.6 Oligonucleotide primers and PCR assay

To validate the presence of amplifiable DNA in all samples, a species-specific PCR targeting the mitochondrial D-loop region producing a fragment of approximately 531 bp was performed using the respective set of forward and reverse primers sequences: FW 5′-AAC CCT ATG TAC GTC GTG CAT-3′ and RV 5′- ACC ATT GAC TGA ATA GCA CCT-3’ (Montiel-Sosa et al., 2000). The PCR reactions were performed in a final volume of 25 μL, containing 2 × NZYTaq II Green Master Mix (NZYtech, Lisboa, Portugal), 0.25 μM of each primer and 20 ng/μL of DNA. A negative and positive control was included in each assay. The reactions were incubated at 95°C for 5 min; followed by 35 cycles of 95°C/30 s, 50°C/30 s, 72°C/30 s, and a final 10 min of extension at 72°C. All runs included internal positive (swine sausage) and negative (fennel leaf) controls. All amplifications steps were performed on a Veriti™ Dx 96-well Thermal Cycler (Thermo Fisher Scientific, Massachusetts, United States). PCR products were separated by electrophoresis in 1.5% agarose gel in 1 × TAE buffer (Tris-acetate-EDTA) (NZYtech, Lisboa, Portugal) stained with Green®Safe Premium nucleic acid stain (NZYtech, Lisboa, Portugal).

### 2.7 Analytical detectability of PCR assay

The detectability of the PCR assay was determined by performing serial dilutions of the DNA extracted from swine sausage and swine hydrolysate and analysing the limit of detection (LOD). To accomplish this, 10-fold dilutions of the DNA extracted ranging from 2 ng to 2 × 10^−6^ ng were tested, where the lowest concentration of DNA that produced a visible PCR product with the expected size was assigned as the detection limit.

## 3 Results and discussion

### 3.1 Integrity, yield, and quality of the extracted DNA

A harmonized DNA extraction protocol is essential not only for obtaining a high yield of high-quality DNA but also for meeting international quality standards, particularly in addressing emerging authenticity issues related to animal by-products. Optimization’s procedures aim to overcome the challenges encountered in PCR detection, particularly inefficient amplification caused by inhibitors substances present in the complex matrices, such as polyphenols, polysaccharides, proteins, lipids, collagen, fulvic acids, among others (Khosravinia and Ramesha, 2007; Mafra et al., 2008; Hedman and Rådström, 2013; Piskata et al., 2017; Sajali et al., 2018). Another constrain of the novel feed ingredients such as meals and hydrolysates is the fact that the DNA molecules have been highly fragmented with the processing stages. Furthermore, the effectiveness of nucleic acid extraction significantly influences the successful amplification of the DNA molecule (Zimmermann et al., 1998; Peano et al., 2004; Mafra et al., 2008).

#### 3.1.1 DNA integrity assessment

**FIGURE 1:**
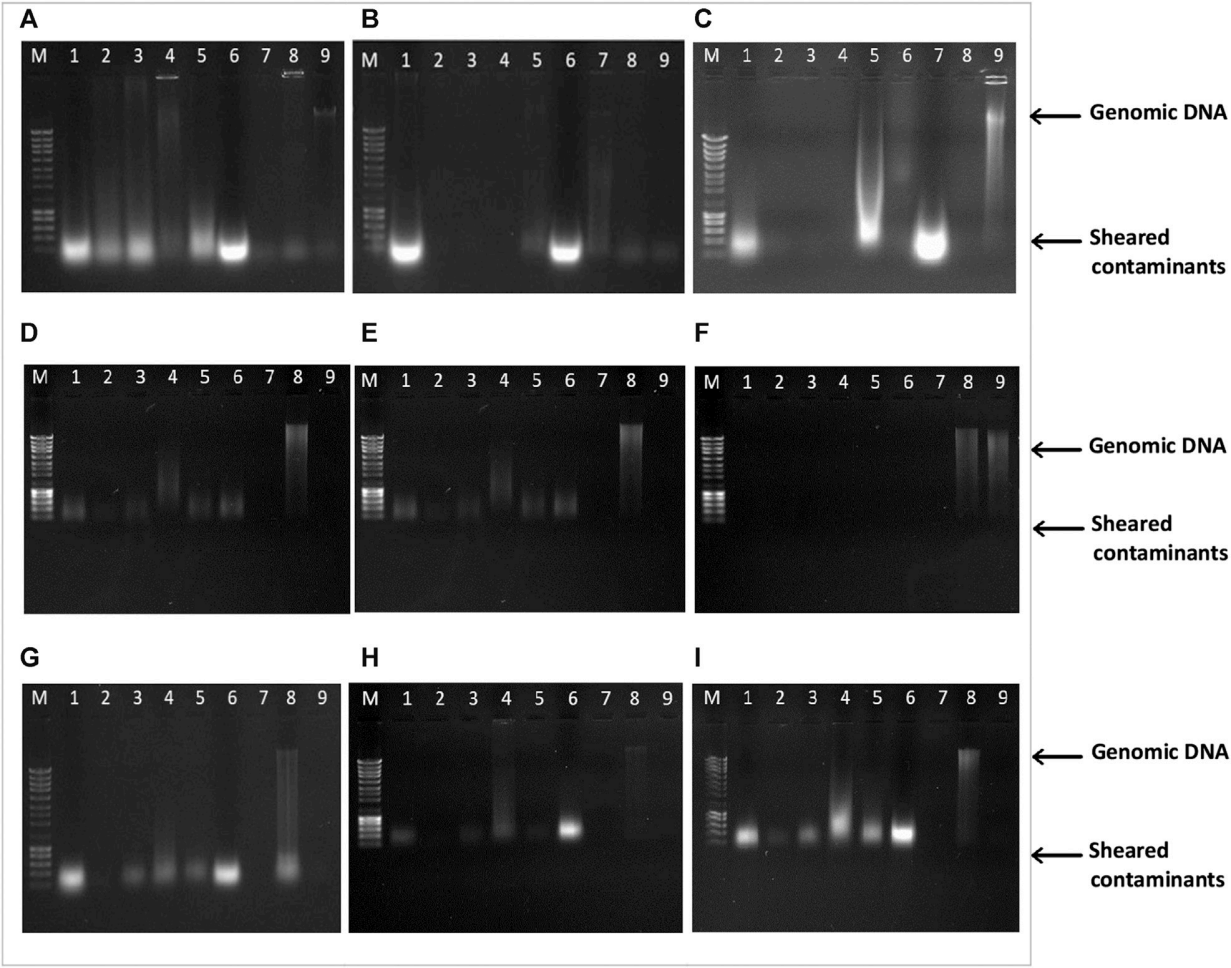
Agarose gel electrophoresis (0.8%) of DNA extracted from Poultry Meat Meal (PMM), Blood (PBM) and Feather meals (FM) using nine different extraction methods: **(A)**-CTAB, **(B)**-CTAB N*, **(C)**-Modified Wizard-CTAB, **(D)**-Modified Wizard-CTAB N*, **(E)**-ZymoBIOMICS™ DNA Miniprep, **(F)**-Quick-DNA™ Miniprep Plus, **(G)**-Invisorb^®^ Spin Tissue Mini, **(H)**-Invisorb^®^ Spin Blood Mini, **(I)**-NucleoSpin™ Food. Lane M- 1 kb DNA ladder; Lane 1- Poultry Meat Meal 1 (PMM1); Lane 2- Poultry Meat Meal 2 (PMM2); Lane 3- Poultry Meat Meal 3 (PMM3); Lane 4- Poultry Meat Meal 4 (PMM4); Lane 5- Poultry Meat Meal 5 (PMM5); Lane 6- Poultry Blood Meal (PBM); Lane 7- Feather Meal (FM); Lane 8- swine sausage (positive control, PC); Lane 9- fennel leaf (negative control, NC).

The selection of the most suitable DNA extraction method directly impacts the success of target amplification. Nine different methods were employed for DNA extraction from novel animal by-products of high interest for the feed industry (Figure 1). The quality of the extracted DNA was initially assessed through electrophoretic analysis. As shown in Figure 1, the observed diffuse bands indicate DNA fragmentation in all samples, likely resulting from elevated temperatures during the heat treatment process, causing a gradual reduction in the size of DNA fragments. Similar findings have been reported in previous studies that attempted DNA isolation from soy and chicken meals using conventional CTAB-based methods and commercial kits (Nesvadbová et al., 2010; Coello et al., 2017). Intense blurring at the terminus of the lanes was noted in both methodologies, which could indicate RNA contamination. This is noteworthy, considering that all samples extracted using both the conventional method (Figures 1A–D) and the NucleoSpin™ Food kit (Figure 1I) underwent treatment with RNase A post-lysis. The presence of smaller DNA fragments may have also contributed to the observed blurring phenomena.

#### 3.1.2 Quality of DNA extracts

The extracted DNA from most samples showed suitable purities for PCR amplification with A260/A280 nm ratio values falling within the optimal range of 1.7–2.0, revealing successful extraction of high purity DNA. However, certain samples, such as Fish hydrolysate (FH) exhibited A260/A280 nm poor ratios (Supplementary Table S1). This fact can be due to the presence of proteins, namely, plasma proteins and antibodies, which absorb light at A280 nm, decreasing the A260/280 nm ratio as reported in previous studies (Olson and Morrow, 2012; Koetsier and Cantor, 2019). In fact, the observed trend in this study was evident in protein rich matrices such as the meal derived from α-keratin and collagen rich feathers (Feather Meal-FM) (Lin et al., 2022), as well as the three protein hydrolysate samples (Meat and Bone Swine Hydrolysate–MBSH; Fish Hydrolysate–- FH, *Black soldier* fly hydrolysate–- BSH) (Supplementary Table S1). Residual impurities persisting from the DNA extraction procedure, such as phenol-chloroform, isoamyl-alcohol and ethanol, have been documented as factors that can reduce the A260/280 nm ratio (Sambrook and Russel, 2001; Wilfinger et al., 2006).

**FIGURE 2:**
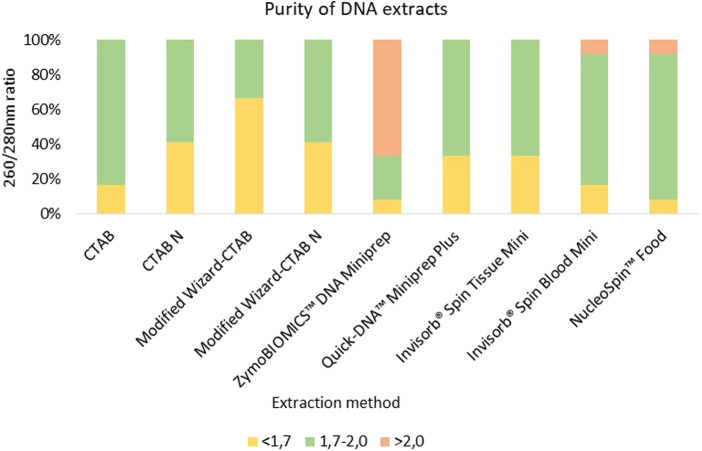
Comparison of DNA extracts purity from 10 meals and hydrolysates derived from animal by-products and two controls samples (swine sausage as positive control and fennel leaf as negative control) employing different extraction methods. DNA extracts with a quality A260/280 nm ratio below 1.7 are colored in yellow, DNA extracts with a quality between of 1.7–2.0 are colored in green, while DNA extracts with A260/280 nm ratio above 2.0 are colored in red.

Conventional methods have proven their effectiveness in extracting high-quality DNA from complex matrices, such as in corn and soy meals (Modified Wizard-CTAB) (Aksoy and Sönmezoğlu, 2022) or honey (CTAB-based method) (Soares et al., 2015). Nonetheless, it´s worth noting that conventional methods are time-consuming in comparison to commercial kits, although they generally yield low-degraded and amplifiable DNA from most food samples (Mafra et al., 2008). In our study, the CTAB-based method (CTAB) showed the highest efficacy in extracting high-quality DNA from all matrices, as indicated by an average A260/A280 nm ratio of 1.78 (Figure 2). Among the evaluated commercial kits, the NucleoSpin™ Food kit emerged as one of the most effective for obtaining high-quality DNA, with an average A260/A280 nm ratio of 1.89 (Figure 2). The NucleoSpin™ Food kit has a well-documented reputation for reliably isolating DNA from processed food and feed products, including soybean meals (Mafra et al., 2008), maize- and soybean-derived food matrices (Peano et al., 2004), and fresh/processed meat samples, including swine (Piskata et al., 2019). The Invisorb^®^ Spin Tissue Mini and Invisorb Spin Blood Mini commercial kits also extracted DNA with average A260/A280 nm ratios exceeding 1.7 (an average A260/A280 nm ratio of 1.77). Conversely, samples extracted using ZymoBIOMICS™ DNA Miniprep kit presented the lowest purity levels, with an average A260/A280 nm ratio of 2.36 (Figure 2). This outcome is likely attributable to the kit’s optimized design for DNA extraction from microbes rather than highly processed matrices.

#### 3.1.3 Yield of DNA extracts

**FIGURE 3:**
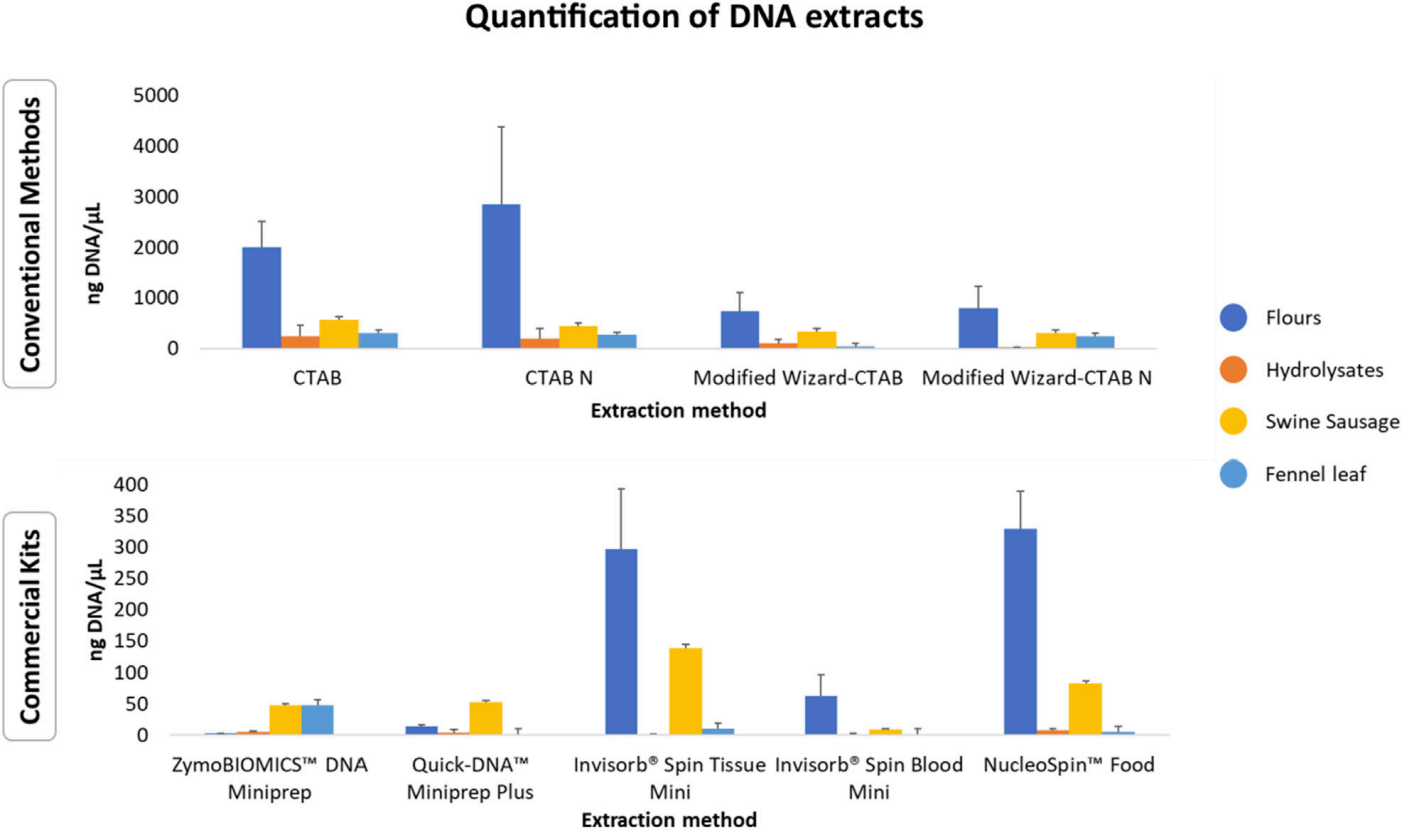
Average DNA concentrations (ng/µL ± standard deviation) of meals and hydrolysates obtained from animal by-products using conventional extraction methods and commercial kits. The data is represented as mean values for each group of matrices.

The concentrations of the extracted DNA from the selected animal processed samples showed significant variability across the tested methods. This is consistent with previous studies indicating that conventional extraction methods generally yield higher amounts of DNA when compared to commercial kits (Figure 3, Supplementary Table S1) (Di Bernardo et al., 2007; Mafra et al., 2008; Piskata et al., 2017). Despite the higher performance of conventional extraction methods regarding DNA yield, it is important to highlight that these methods are prone to contamination by chemical reagents during the extraction process. For instance, the presence of residual CTAB may increase the DNA solution’s absorbance at A260 nm and thus result in increased values of obtained concentration (Drábková et al., 2002; Piskata et al., 2019; Schenk et al., 2023). This may occur due to less efficient purification steps when compared to commercial kits that applied silica-based columns, which enhance impurity removal, although lower yields are obtained (Shetty, 2020).

Regardless of the extraction method used, the technological processes applied to the selected animal meals and hydrolysates, such as mechanical treatment, high temperature, high pressure, autohydrolysis, enzymatic hydrolysis, can significantly impact both DNA yield and integrity. This variability can be attributed to the potential of these processes to induce DNA fragmentation, cross-linking with other compounds, or denaturation (Bauer et al., 2003; Bergerová et al., 2010; Şakalar et al., 2012). Among all the samples tested, meals yielded the highest amount of DNA (Figure 3; Supplementary Table S1). Overall, the Poultry Meat Meal (PMM) samples and the Poultry Blood Meal (PBM) sample exhibited the highest yields, while the Feather Meal (FM) samples had the lowest yields (Supplementary Table S1). These variations could be due to the elevated keratin content in feathers, requiring extraction protocols that break down the keratin for DNA liberation (Lin et al., 2022). This process is accomplished by employing digestion buffers rich in detergents and reducing agents (e.g., SDS, DTT, or Cleland’s reagent), along with proteinase K, as utilized in the CTAB-based method (933 ng/μL ± 4.83) and NucleoSpin™ Food kit (145.11 ng/μL ± 17.76). Regarding the analysis of hydrolysates, most of the extraction methods resulted in low DNA yields, except for *Black soldier* fly Hydrolysate (BSH) extracted with the conventional methods CTAB-based method (CTAB), Modified CTAB-based method (CTAB N*) and Modified Wizard-CTAB (Supplementary Table S1). The obtained results are in some manner expected, as these ingredients are known for their high protein content (>60%) (Chalamaiah et al., 2012; Resende et al., 2022).

### 3.2 PCR detectability evaluation

**FIGURE 4:**
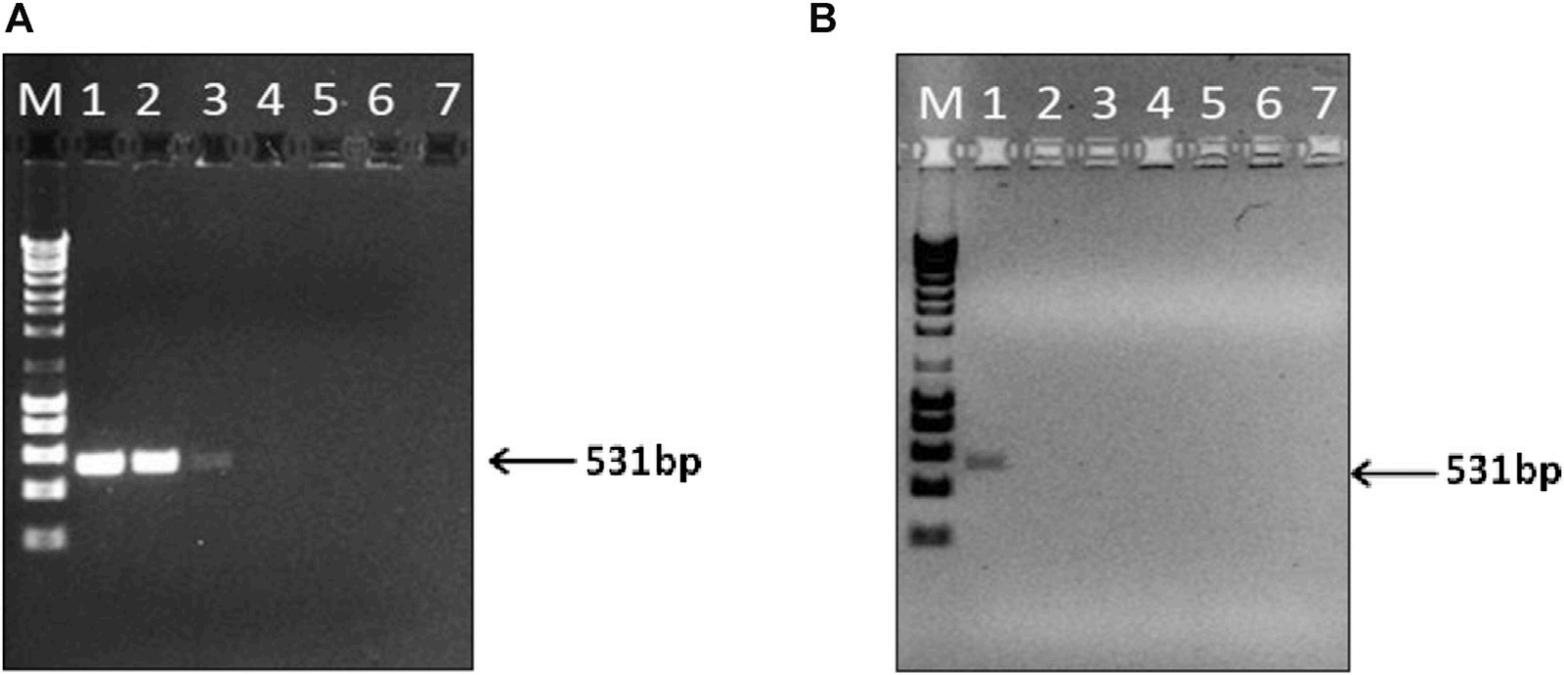
Limit of detection (LOD) of the PCR assay for the detection of swine DNA extracted from swine sausage (positive control) **(A)**, and Meat and Bone Swine Hydrolysate (MBSH) **(B)**. PCR products amplified with serial 10-fold dilutions of extracted DNA ranging from 2 ng (lane 1) to 2 × 10^−6^ ng of total DNA (lane 7). M - DNA ladder of 1 kb.

To guarantee that PCR amplifications between different DNA extractions were reproducible and reliable to detect the presence of swine DNA, a species-specific PCR targeting the mitochondrial (mtDNA) D-loop of swine DNA was used (Figure 4). This non-coding region was selected for its presence in degraded samples, its stability, and its lower susceptibility of recombination compared to nuclear DNA (Luo et al., 2011; Ali et al., 2012; Yin et al., 2020), allowing to successfully detect swine DNA in processed food samples as the ones tested in this study (Montiel-Sosa et al., 2000; Karabasanavar et al., 2014; Sepminarti et al., 2016).

In this study, the PCR detectability was significantly lower (100-fold) for hydrolysate samples (2 ng) than that obtained for sausage samples, which attained LOD values of 20 pg (Figures 4A, B). Similar sensitivities levels were achieved in other studies using the mitochondrial D-loop as target for identifying swine DNA in food samples using commercial extraction kits (Karabasanavar et al., 2014; Sepminarti et al., 2016). Notably, Lin et al. (2022) achieved an LOD of 10 fg for swine meat employing the CTAB-based method, even though targeting the mitochondrial *cytb* gene.

### 3.3 Assay validation and authenticity of novel feed ingredients

**FIGURE 5:**
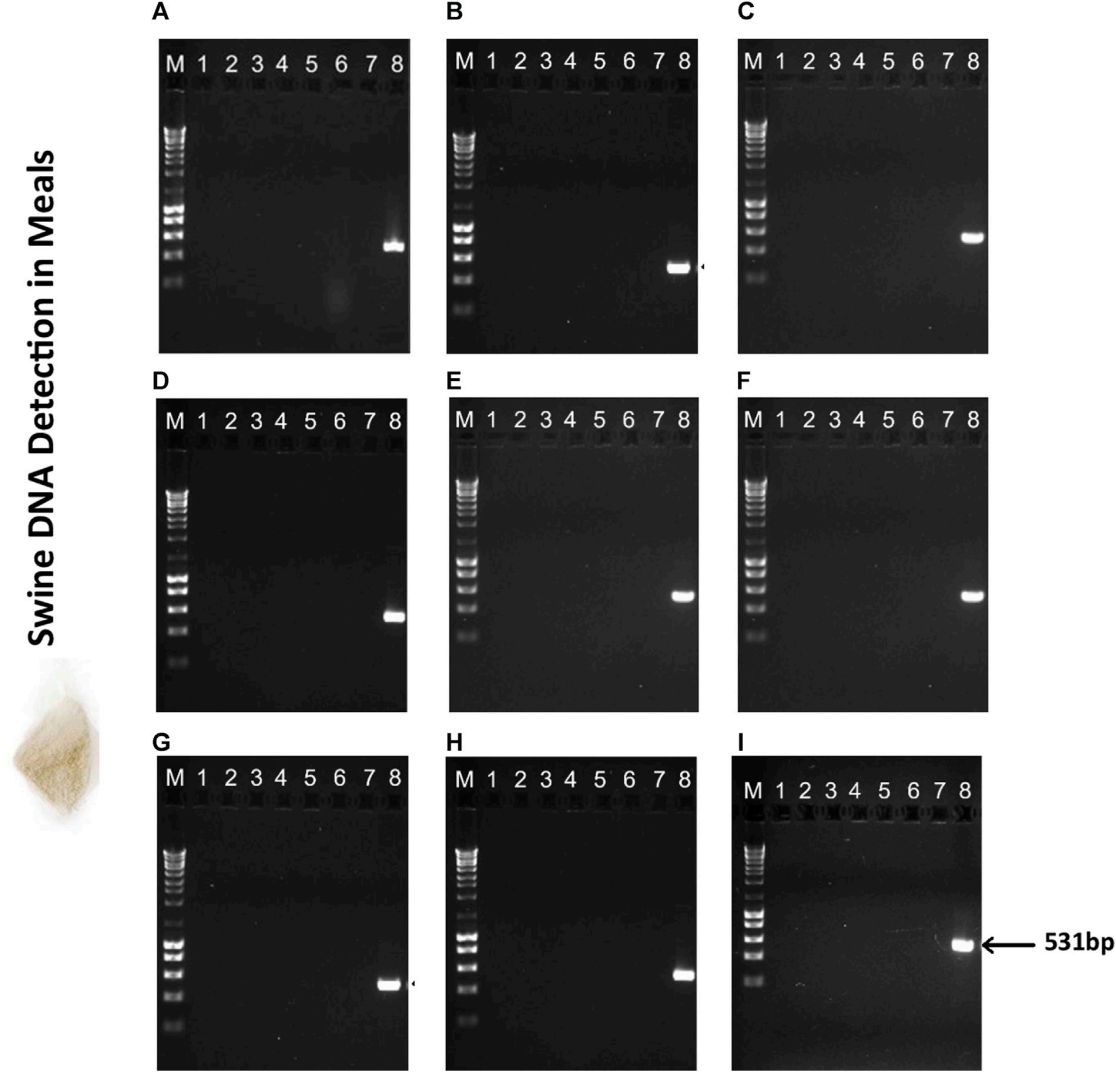
Agarose gel electrophoresis (1.5%) of PCR assay targeting the swine mitochondrial D-loop amplicon of 531 bp in meals used in aquafeed production. **(A)** CTAB, **(B)** CTAB N*, **(C)** Modified Wizard-CTAB, **(D)** Modified Wizard-CTAB N*, **(E)** ZymoBIOMICS™ DNA Miniprep, **(F)** Quick-DNA™ Miniprep Plus, **(G)** Invisorb^®^ Spin Tissue Mini, **(H)** Invisorb Spin Blood Mini, **(I)** NucleoSpin Food. Lane M- 1 kb DNA ladder; Lane 1- Poultry Meat Meal 1 (PMM1); Lane 2- Poultry Meat Meal 2 (PMM2); Lane 3- Poultry Meat Meal 3 (PMM3); Lane 4- Poultry Meat Meal 4 (PMM4); Lane 5- Poultry Meat Meal 5 (PMM5); Lane 6- Poultry Blood Meal (PBM); Lane 7- Feather Meal (FM); Lane 8- swine sausage (positive control, PC).

**FIGURE 6:**
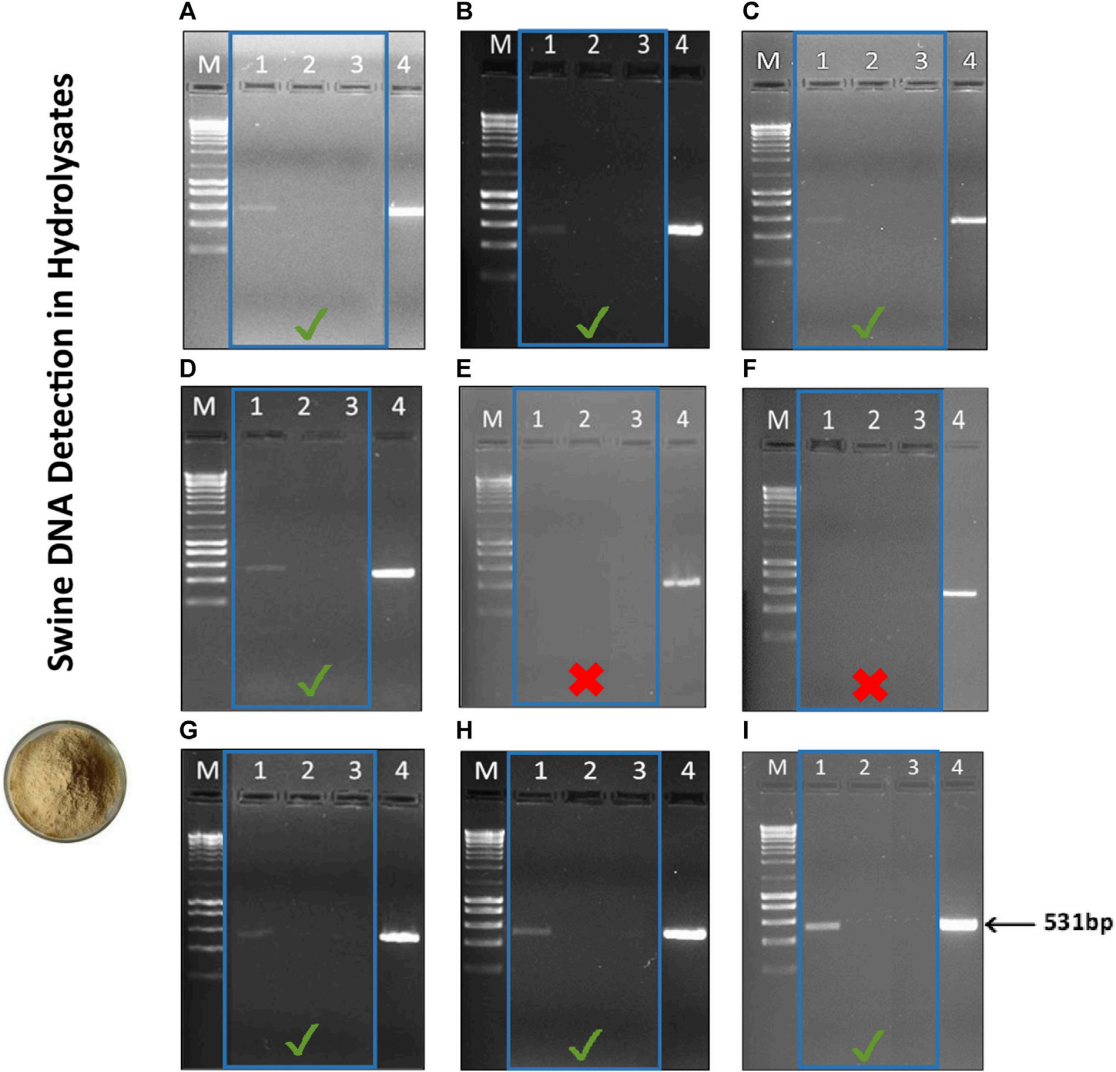
Agarose gel electrophoresis (1.5%) of PCR assay targeting the swine mitochondrial D-loop amplicon of 531 bp in hydrolysates used in aquafeed production. **(A)** CTAB, **(B)** CTAB N*, **(C)** Modified Wizard-CTAB, **(D)** Modified Wizard-CTAB N*, **(E)** ZymoBIOMICS™ DNA Miniprep, **(F)** Quick-DNA™ Miniprep Plus, **(G)** Invisorb^®^ Spin Tissue Mini, **(H)** Invisorb Spin Blood Mini, **(I)** NucleoSpin Food. Lane M- 1 kb DNA ladder; Lane 1- Meat and Bone Swine Hydrolysate (MBSH); Lane 2- Fish Hydrolysate (FH); Lane 3- Black soldier fly Hydrolysate (BSH); Lane 4- Swine sausage (positive control, PC). The PCR results obtained for the different hydrolysates are highlighted by the blue square.

The 10 meals and hydrolysates obtained from animal by-products were evaluated to ensure compliance with the halal market regarding the presence of swine DNA (Figures 5, 6). As expected, in accordance with the labelled information, the MBSH sample exhibited a positive result for swine in the qualitative PCR for most of the assessed extraction methods (Figure 6). The sole exception was the MBSH extracted using the ZymoBIOMICS™ DNA Miniprep and Quick-DNA™ Miniprep Plus commercial kit (Figure 6).

The amplified DNA fragment from the MBSH sample had an estimated size of 531bp and there were no impurities detected, such as contamination, product degradation or primer-dimer. The PCR analysis confirmed the absence of swine DNA in the remaining meal (Figure 5) and hydrolysate samples (Figure 6), indicating that these ingredients did not contain any swine derivatives in their composition, confirming the labelled information.

## 4 Conclusion

**FIGURE 7:**
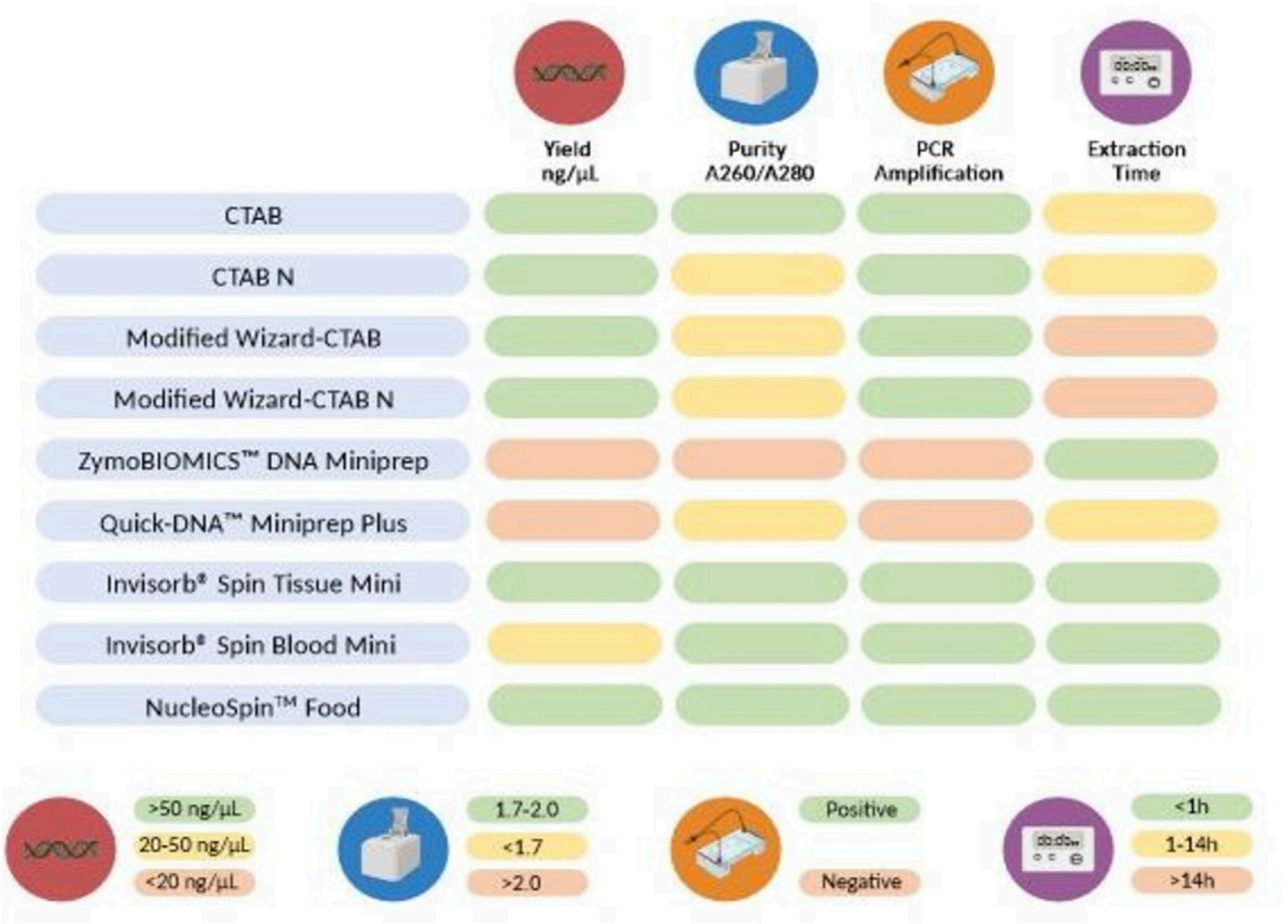
Parameters employed to compare the nine DNA extraction methods. The color code represent the classification of each method’s outcome for each parameter: green (good), yellow (medium), or red (poor). Average Yield per extraction method: <20 ng/μL (red), 20–50 ng/μL (yellow), >50 ng/μL (green); Average A260/280 nm ratio: >2.0 (red), <1.7 (yellow), 1.7–2.0 (green); PCR amplification: successful amplification of swine DNA on samples containing swine (green), no amplification of swine DNA in swine containing samples (red); Average extraction time: >10 h (red), 1–10 h (yellow), <1 h (green).

In this study, we conducted a thorough evaluation of various DNA extraction methods to determine the most effective protocol for isolating DNA, specifically for the detection of swine in commercial feed, as schematized in Figure 7. Most of the DNA extraction methods (conventional and commercial kits) demonstrated the ability to extract high-quality DNA that could be effectively amplified by PCR, as evidenced by the presence of a 531 bp fragment in the MBSH. While the conventional protocols produced the highest amount of DNA, they were also the most time-consuming, requiring an average of 14–16 h to complete the protocol. Among these methods, the CTAB-based approach (CTAB) proved to be the most proficient in terms of both DNA yield and quality making it suitable for PCR-based approaches. The present results also evidenced the superiority of the NucleoSpin™ Food and Invisorb^®^ Spin Tissue Mini in extracting DNA from meals and hydrolysates from animal by-products. This method offers the advantage of simplicity and speed while consistently providing high-quality DNA. Overall, the findings indicate that most of the tested extraction protocols were successful in extracting and identifying swine DNA from complex matrices and highly processed samples and that the choice of method in assessing the authenticity of novel feed ingredients should consider various factors such as time, cost, and the specific objectives of the study.

## Data Availability

The data that support the findings of this study are available from the corresponding author upon reasonable request.
