# Supplementary figures and images for: Enhancing the authenticity of animal by-products: harmonization of DNA extraction methods from novel ingredients
Source: Front Chem. 2024 Feb 20;12:1350433. doi: 10.3389/fchem.2024.1350433 (PMC10912508; doi:10.3389/fchem.2024.1350433)

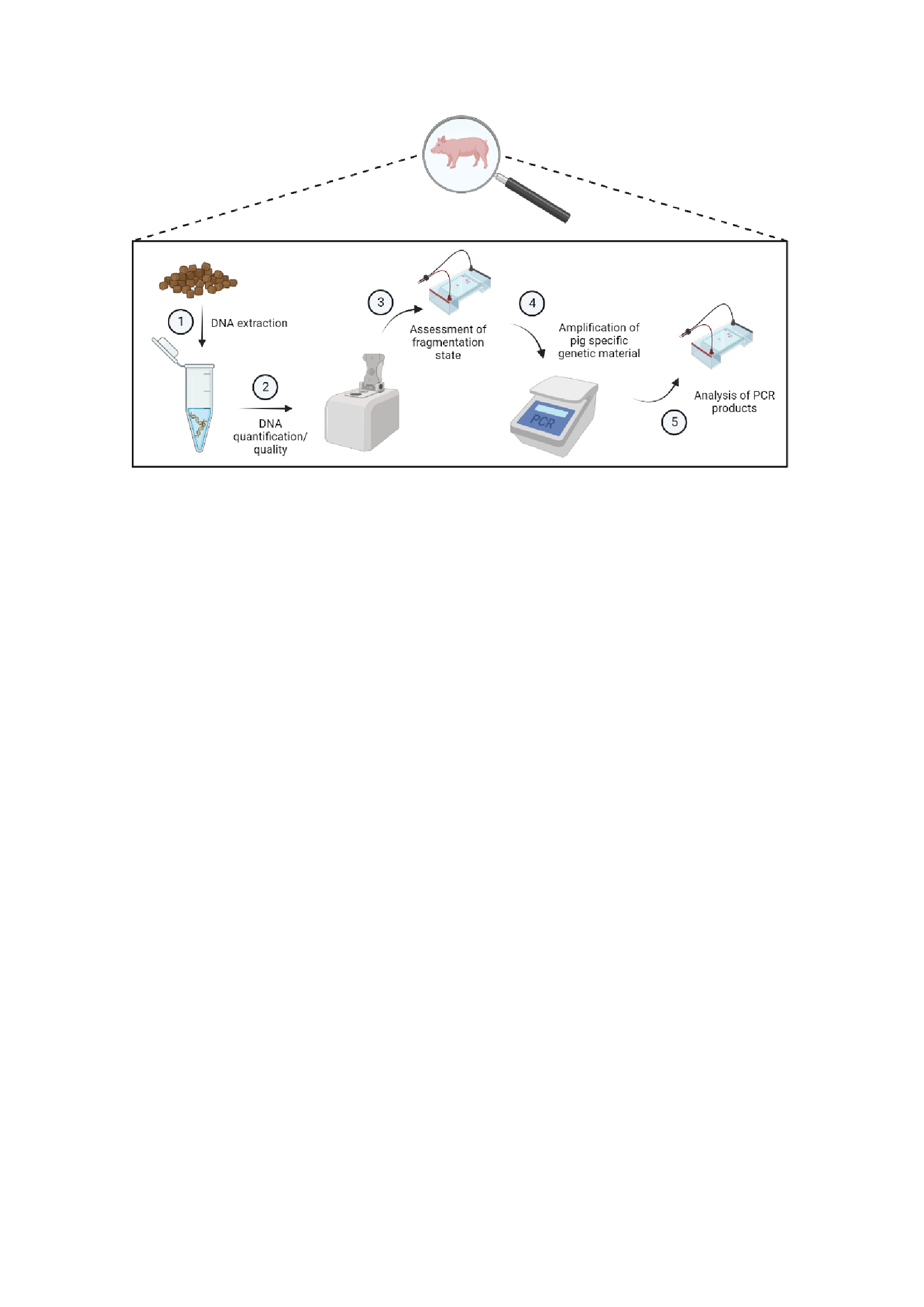

Supplement: Supplementary file 1 [file Image1.TIFF]
