# Supplementary material for: Enhancing the authenticity of animal by-products: harmonization of DNA extraction methods from novel ingredients
Source: Front Chem. 2024 Feb 20;12:1350433. doi: 10.3389/fchem.2024.1350433 (PMC10912508; doi:10.3389/fchem.2024.1350433)
Supplement: Supplementary file 2 [file DataSheet1.docx]

| **Supplemental Table 1.** Average values obtained from the DeNovix reads relatively to the DNA yield, purity and extraction time for novel aquafeed ingredients extracted with conventional methods. DNA concentrations are expressed in ng/µL. The extraction time was calculated as the total time needed to complete the extraction protocol. | | | | |
| --- | --- | --- | --- | --- |
| **Extraction method** | **Sample** | **Average Concentration (ng/µL)** **± SD** | **A260/A280** | **Extraction time** |
| CTAB-based method (CTAB) | PMM1 | 4363.00± 181.87 | 1.92 | 14h |
|  | PMM2 | 1346.00 ±28.78 | 1.86 |  |
|  | PMM3 | 2396.00 ±169.88 | 1.88 |  |
|  | PMM4 | 703.60 ±77.79 | 1.99 |  |
|  | PMM5 | 1082.00 ± 67.46 | 1.93 |  |
|  | PBM | 3120.00 ± 92.15 | 1.87 |  |
|  | FM | 933.00 ± 4.83 | 1.73 |  |
|  | MBSH | 8.43 ± 1.38 | 1.54 |  |
|  | FH | 19.78 ± 1.86 | 1.45 |  |
|  | BSH | 686.10 ± 50.70 | 1.72 |  |
|  | PC | 569.70 ± 11.58 | 1.72 |  |
|  | NC | 303.20 ± 12.29 | 1.71 |  |
| CTAB-based modified method (CTAB N)* | PMM1 | 9452.40 ± 434.95 | 1.99 | 14h |
|  | PMM2 | 10.37 ± 10.86 | 1.63 |  |
|  | PMM3 | 12.39 ± 4.47 | 1.81 |  |
|  | PMM4 | 1818.68 ± 56.08 | 1.76 |  |
|  | PMM5 | 560.73 ± 30.06 | 1.75 |  |
|  | PBM | 7943.63 ± 31.61 | 1.93 |  |
|  | FM | 95.60 ± 2.92 | 1.52 |  |
|  | MBSH | 8.39 ± 2.42 | 1.11 |  |
|  | FH | 11.14 ± 1.80 | 1.17 |  |
|  | BSH | 576.61 ± 30.50 | 1.62 |  |
|  | PC | 437.60 ± 18.65 | 1.77 |  |
|  | NC | 264.49 ± 5.04 | 1.76 |  |
|  |  |  |  |  |
| Modified Wizard-CTAB | PMM1 | 447.30 ± 106.92 | 1.77 | 16h |
|  | PMM2 | 72.70 ± 9.84 | 1.41 |  |
|  | PMM3 | 18.50 ± 2.47 | 1.41 |  |
|  | PMM4 | 1922.60 ± 136.04 | 1.86 |  |
|  | PMM5 | 234.60 ± 8.76 | 0.97 |  |
|  | PBM | 2409.40 ± 232.81 | 1.85 |  |
|  | FM | 26.50 ± 6.41 | 1.53 |  |
|  | MBSH | 11.51 ± 2.47 | 1.50 |  |
|  | FH | 6.95 ± 3.81 | 1.43 |  |
|  | BSH | 275.15 ± 30.40 | 1.71 |  |
|  | PC | 332.90 ± 70.31 | 1.39 |  |
|  | NC | 46.10 ± 7.41 | 1.55 |  |
|  |  |  |  |  |
| Modified Wizard-CTAB N* | PMM1 | 2937.43 ± 141.42 | 1.91 | 16h |
|  | PMM2 | 32.96 ± 2.83 | 1.64 |  |
|  | PMM3 | 6.15 ± 1.41 | 1.69 |  |
|  | PMM4 | 1616.47 ± 162.49 | 1.82 |  |
|  | PMM5 | 4.14 ± 1.41 | 2.04 |  |
|  | PBM | 201.47 ± 23.33 | 1.82 |  |
|  | FM | 806.22 ± 20.51 | 1.86 |  |
|  | MBSH | 35.16 ± 9.19 | 1.02 |  |
|  | FH | 3.13 ± 1.41 | 0.71 |  |
|  | BSH | 22.88 ± 9.12 | 1.71 |  |
|  | PC | 307.30 ± 21.32 | 1.72 |  |
|  | NC | 247.00 ± 17.66 | 1.20 |  |
| \| * N - Initial grinding of the samples with a mortar and pestle in liquid nitrogen  SD – Standard Deviation \| \| --- \| | | | | |
|  |  |  |  |  |

| **Supplemental Table 2.** Average values obtained from the DeNovix reads relatively to the DNA yield, purity and extraction time for novel aquafeed ingredients extracted with commercial kits. DNA concentrations are expressed in ng/µL. The extraction time was calculated as the total time needed to complete the extraction protocol. | | | | |
| --- | --- | --- | --- | --- |
| **Extraction method** | **Sample** | **Average Concentration**  **(ng/µL)** **± SD** | **A260/A280** | **Extraction time** |
| ZymoBIOMICS™ DNA Miniprep | PMM1 | 3.55 ± 0.61 | 2.23 | 15min |
|  | PMM2 | 2.56 ± 0.06 | 1.88 |  |
|  | PMM3 | 0.73 ± 0.88 | 1.76 |  |
|  | PMM4 | 0.48 ± 0.21 | 1.94 |  |
|  | PMM5 | 3.20 ± 0.45 | 1.31 |  |
|  | PBM | 2.86 ± 0.29 | 4.64 |  |
|  | FM | 3.13 ± 1.07 | 2.15 |  |
|  | MBSH | 4.01 ± 1.52 | 2.74 |  |
|  | FH | 4.35 ± 0.56 | 3.08 |  |
|  | BSH | 8.60 ± 1.78 | 2.20 |  |
|  | PC | 47.35 ± 2.10 | 2.19 |  |
|  | NC | 47.85 ± 3.24 | 2.13 |  |
|  |  |  |  |  |
| Quick-DNA™ Miniprep Plus | PMM1 | 19.38 ± 0.66 | 1.76 | 1-3h |
|  | PMM2 | 5.19 ± 1.41 | 1.69 |  |
|  | PMM3 | 9.85 ± 1.76 | 1.90 |  |
|  | PMM4 | 25.67 ± 4.74 | 1.98 |  |
|  | PMM5 | 14.33 ± 1.41 | 1.76 |  |
|  | PBM | 19.68 ± 0.66 | 1.93 |  |
|  | FM | 0.90 ± 0.21 | 1.42 |  |
|  | MBSH | 0.51 ± 0.15 | 0.49 |  |
|  | FH | 0.57 ± 0.10 | 1.23 |  |
|  | BSH | 12.44 ± 2.90 | 1.88 |  |
|  | PC | 52.29 ± 11.83 | 1.94 |  |
|  | NC | 1.16 ± 1.48 | 1.28 |  |
|  |  |  |  |  |
| Invisorb® Spin Tissue Mini | PMM1 | 732.98 ± 38.71 | 1.96 | 1h |
|  | PMM2 | 15.96 ± 1.61 | 1.74 |  |
|  | PMM3 | 99.64 ± 7.49 | 1.84 |  |
|  | PMM4 | 125.13 ± 3.61 | 1.97 |  |
|  | PMM5 | 110.36 ± 8.50 | 1.77 |  |
|  | PBM | 990.49 ± 5.47 | 1.97 |  |
|  | FM | 5.70 ± 0.85 | 1.59 |  |
|  | MBSH | 0.16 ± 0.05 | 1.46 |  |
|  | FH | 0.10 ± 0.03 | 1.63 |  |
|  | BSH | 2.58 ± 0.69 | 1.82 |  |
|  | PC | 138.42 ± 27.00 | 1.96 |  |
|  | NC | 10.39 ± 1.06 | 1.55 |  |
| Invisorb® Spin Blood Mini | PMM1 | 38.26 ± 7.07 | 1.84 | 20min |
|  | PMM2 | 16.77 ± 2.45 | 1.61 |  |
|  | PMM3 | 22.47 ± 1.52 | 1.68 |  |
|  | PMM4 | 81.37 ± 7.05 | 1.91 |  |
|  | PMM5 | 12.11 ± 2.68 | 1.74 |  |
|  | PBM | 259.65 ± 14.55 | 1.89 |  |
|  | FM | 3.76 ± 1.09 | 1.80 |  |
|  | MBSH | 4.76 ± 0.93 | 1.85 |  |
|  | FH | 0.44 ± 0.23 | 1.68 |  |
|  | BSH | 0.11 ± 0.01 | 1.21 |  |
|  | PC | 9.55 ± 2.82 | 1.85 |  |
|  | NC | 0.99 ± 0.15 | 2.15 |  |
|  |  |  |  |  |
| NucleoSpin^TM^ Food | PMM1 | 257.18 ± 14.14 | 1.88 | 66min |
|  | PMM2 | 23.64 ± 7.07 | 1.81 |  |
|  | PMM3 | 111.48 ± 21.21 | 1.92 |  |
|  | PMM4 | 718.81 ± 42.76 | 1.95 |  |
|  | PMM5 | 208.17 ± 6.95 | 1.91 |  |
|  | PBM | 840.74 ± 52.61 | 1.97 |  |
|  | FM | 145.11 ± 17.76 | 1.92 |  |
|  | MBSH | 8.28 ± 0.74 | 1.83 |  |
|  | FH | 1.18 ± 0.13 | 2.46 |  |
|  | BSH | 12.33 ± 2.11 | 1.63 |  |
|  | PC | 82.38 ± 4.90 | 1.75 |  |
|  | NC | 5.53 ± 1.61 | 1.65 |  |

SD – Standard Deviation
